# Professional Pressure

**Published:** 1889-11-02

**Authors:** 


### The Doctor's Pulpit.

### PROFESSIONAL PRESSURE.

Mr. Darwin frequently expressed the conviction that the "struggle for existence" is of great value to the strugglers, and that, being a necessary factor in the development of human beings as well as of other animals, it is decidedly likely to continue. If this be so, then the general practitioners of medicine, and a good many who object to be classed with general practitioners, will surely in no long time become very highly developed organisms and intellectualities. For the "existence" of a considerable proportion of our professional *confrères* is practically "all struggle." The modern medical man stakes his soul on science and development. It is therefore incumbent upon him to stand loyally by his own principles. If it be the fact that "struggle" is the scientific law of human development, then the doctor, who is the student and expounder of the law, ought to be the last man in the world to complain of what is really and truly a principal article in his scientific creed.

There are not wanting indications, however, in the medical journals and elsewhere, that the average medical man would rather get his knowledge of "development" and "the struggle for existence" from books than acquire it by personal and practical experience. Even Darwin probably preferred to think of the conflicts and strifes of prehistoric mammals, or at any rate of the contests of male lions, or of seals, for the possession of the most pleasing wives, to engaging in a hand-to-hand battle with life's difficulties on his own account. Such, unhappily—or, perhaps, happily—is the unwillingness of man to fight if he can help it.

The real truth about the average man, be he doctor or otherwise, seems to be that he hates life to be made too hard for him. A moderate exercise of his intellectual faculties, a reasonable degree of bodily exertion, a fair proportion of difficulty and danger, he does not object to. But when life presents too many problems for solution, and most of them of too difficult a kind; when cares and toils occupy not only the working hours, but also those that should be exclusively devoted to food, and sleep, and play, then the doctor begins to think that there is a little too much of the "struggle for existence" accompaniment to his life-song. Perhaps he is right! But if such struggles be only the application of universal principles to his own particular case, it becomes rather a nice question in morals how far he is justified in interfering with those general conditions which are the very essentials of his own upward progression into the higher regions of scientific illumination. Let no profane reader cry "rubbish"! Everybody knows quite well that a scientist, like every other idolater, is bound to obey the divinities of his own creation. Is it not familiar to all men that political economists laboured for generations to construct a science, whole, round and complete; and that when they had constructed and finished their fetish no dog nor even man was, for many years, permitted to wag his tongue against the lightest word of the dismal deity? When Mr. Gladstone, a few years ago, profanely relegated some of the doctrines of Adam Smith's descendants to Saturn, it was felt that no bolder impiety than his was possible to modern man.

There are, however, a good many medical men who either have not the clearness of intellect to perceive that strict obedience to the laws involved in the universal struggle for existence is the only logical outcome of their particular scientific position, or they have so much faith in that discredited divinity of unscientific generations called "common-sense" that they are actually both willing and anxious to abate the severity of the struggle for existence by all the means in their power. We confess to a profound sympathy with these scientific heretics and reprobates. If the toilworn and ill-paid doctor can by any device get an easier and a better time of it, we should be glad to see him do so, notwithstanding that there might thus be occasioned a serious interference with the action of Nature's developmental forces. It is certainly a dreadful thing to set up any opposition whatever to the rigid and inflexible law and gospel of science, however proper and right from a scientific point of view it may be to be sceptical as to the law and the gospel of religion. Nevertheless, in the interests of a large and not undeserving class, who seem to be too hard pressed in the struggle for existence, we venture to take the side of the heretics who are minded to make the peas in the shoes of the medical pilgrim a little less hard, or even to submit them to a process of gentle boiling.

In what direction, if any, is hope of relief for the hardly-pressed medical man to be looked for? It is a familiar principle of his daily professional life to look for the causes of the ills he is called upon to cure. What, then, may be the causes of the modern medical man's want of a reasonably endurable lot in life. "*Divide et impera*," divide and rule, has always been the bane of the medical profession. Doctors have not waited for outside forces to divide and disintegrate them. They have divided and disintegrated themselves. The public has thus had an easy victory; and now has a large portion of the medical profession completely at its mercy. Does anyone suppose, for example, that a medical man would take clubs at five shillings a year per man, or that after a hard day's work he would have an evening consulting hour if he could help it; or that he would turn out of his comfortable bed at night except in cases of real emergency, if he dared to refuse? But he knows that in many parts of London and other large towns he may as well take down his doorplate and send for the furniture van at once as refuse to see patients in the evening, or decline a five shillings club. Many of those private patients who visit him between eight and ten p.m. could just as well call upon him before his morning round; and if the doctor were in a more independent position they would have to do so. But where is the general practitioner, even in the richer districts of London, who dares to announce that his consulting-room will on no account be opened, except for an emergency, after seven o'clock in the evening? If all would agree to do it the difficulty would be over; and no doctor would be one whit the poorer, nor any patient injured in the least. But if one were to close, a hundred others would the more eagerly remain open, and would be only too glad to get an additional half-crown each from his scattered clients. It is of no use for doctors to complain. They have the remedy in their own hands. That remedy is combination for mutual safety. So long as they deliberately elect to be jealous of and to devour one another the only thing that ought to be said is, "It serves you rght, gentlemen, to be devoured."

All classes of operatives in this country have learnt the art of combination, and have steadily and surely improved their position as a result. Do doctors think it "undignified" to combine like workmen? They may be reminded that peers and other landed proprietors have found it desirable to combine; farmers also have followed in their wake; clergymen, too, combine for defence; and lawyers apparently cannot exist without combination and mutual help. Is there any more reason why medical men should not combine than why peers should not? It is not really a sense of dignity that keeps doctors apart, but miserable mutual distrust and selfishness.

And yet doctors are not worse than other men, or more distrustful and selfish. They have inherited bad traditions. That is really the principal reason of their separateness and apparent sordidness. Tradition dies hard; how hard, only those know who have sometimes found themselves seriously and conscientiously opposed to it. But the medical man of the present time is a very different person from those professional ancestors to whose traditions he so stedfastly clings. Is it not more than unworthy that the university graduate and the carefully-taught hospital student of to-day should maintain and strive to perpetuate the unworthy and often worse than vulgar traditions of the apothecary and the barber-surgeon of a period long gone by? The combination of medical men for mutual protection and help can do nothing but good to themselves, both socially, financially, and professionally; and it can do no harm to the public in any way, but, on the contrary, only good. For whatever raises the status and prosperity of the doctor tends to make him more capable and trustworthy as a practitioner and more honourable as a man. There are a good many schemes on foot at the present time for promoting the union and the greater prosperity of the profession; but none of them can succeed unless they are taken up by individuals with honest intent and enthusiastic purpose. To make life as tolerable and as enjoyable for the doctor as it is for the lawyer and the clergyman, is an object not unworthy of the support of every member of the profession, even of those whose prosperity is assured and whose lines have fallen to them in the pleasant places of well-paid consulting practice. The whole profession constitutes one single body, and it can never be well with the body as a whole whilst the greater part of the members are overworked, underpaid, and a prey to discontent and disgust.

